# Editorial: New Advances in Non-small Cell Lung Cancer Management: Immune Modulation and Targeted Therapies

**DOI:** 10.3389/fmed.2021.761078

**Published:** 2021-11-25

**Authors:** Thierry Berghmans, Jacques Cadranel, Bogdan Grigoriu

**Affiliations:** ^1^Clinic of Thoracic Oncology, Institut Jules Bordet, Université Libre de Bruxelles, Brussels, Belgium; ^2^Assistance Publique Hôpitaux de Paris, Hôpital Tenon and Groupes de recherche clinique Theranoscan and Curamus Sorbonne Université, Paris, France; ^3^Service des Soins Intensifs et Urgences Oncologiques, Institut Jules Bordet, Université Libre de Bruxelles (ULB), Brussels, Belgium

**Keywords:** lung, immunotherapy, targeted therapy, PD1, PDL1

Lung cancer is a common pathology with poor prognosis, with non-small cell histologies accounting for 85% of the diagnoses. Most of the patients require exposure to a systemic therapy (1). During the twentieth century, some survival advances were obtained by introducing chemotherapy (CT), initially with platinum derivatives and thereafter with third generation agents (pemetrexed, gemcitabine, or vinorelbine) (2). However, treatment results are still unsatisfactory and do not match those of other cancers. Continuous research developments led to two revolutions during the twenty-first century.

DNA instability is a hallmark of cancer conferring a survival advantage and metastatic capabilities to tumors cells, lung cancer being one of the tumors with the highest rate of mutations. The discovery of EGFR mutations and their relationship with response to TKI inhibitors generated a strong clinical interest. EGFR positive tumors are susceptible to small inhibitory molecules targeting the mutated tyrosine kinase domain of the EGF receptor (EGFR TKI) (3). Dramatic improvements have been reported in randomized clinical trials comparing EGFR TKI to CT in stage IV diseases (4). Resistance to TKIs is the rule, despite its improved evolution and tolerance compared to CT, due to various so-called escape mechanisms, such as secondary mutations on the same or on parallel signaling pathways (T790M, MET, or KRAS mutation) or histological transformation (small-cell lung cancer). Second and third generation TKIs were developed, offering either better efficacy in first-line therapy or salvage treatment of secondary mutations (osimertinib for T790M secondary mutation) (5). Other activable oncogenic driver mutations (BRAFV600 or KRAS G12C) or translocations (ALK, ROS1, RET, or NTRK) are continuously described and new therapies (generally oral agents) are added to the treatment armamentarium.

The evolving knowledge in the landscape of tumor mutations required major changes in routine practice. Pathological examination is not only needed for diagnosis but mandates subtype assessment both at histologic (squamous vs. non-squamous) and molecular levels in order to guide the practitioner through diagnostic algorithms that are continuously adapted (6). We need to provide adequate tumor samplings in terms of quantity and quality to the pathologist who has to choose the most appropriate laboratory investigations. We evolved from single EGFR PCR 10 years ago, to high-throughput sequencing technologies both at the DNA and mRNA levels. The complexity and the evolution of this diagnostic strategy is stressed in the first article of this series (Domagala-Kulawik).

The second revolution occurred later in the twenty-first century. For years, great effort was put into immunotherapy for cancer treatment, generally with little or no clinical efficacy. The discovery of immune checkpoints gave a second boost to immunotherapy in lung cancer. Immune checkpoint inhibitors targeting programmed death-1 (PD1), programmed death-ligand-1 (PDL1), or cytotoxic T-Lymphocyte Associated 4 (CTLA4) rapidly demonstrated their robust interest in phase I-II studies and phase III trials turned positive for anti PD1 and antiPDL1 antibodies with a favorable toxicity profile (7, 8) and a survival plateau after 5 years' follow-up. In 2021, standard first-line therapy for advanced and metastatic non-small cell lung cancer (NSCLC) includes immunotherapy, with or without chemotherapy, in the absence of oncogenic driver. Immunotherapy has become a standard after radio-chemotherapy in stage III NSCLC almost doubling the percentage of long-term survivors. Its role after surgery should be determined in the following years after obtaining the final data from phase III trials. Evolution of the therapeutic strategies and evidence of immunotherapy effectiveness are the subjects of the second manuscript of the series (Berghmans et al.).

Despite better efficacy and tolerance than CT alone, only a limited number of patients have a long-lasting benefit (more than 18 months) from immunotherapy. Moreover, this is a very expensive treatment, and the increasing cost and number of new therapies raises the question of long-term sustainability of this approach. Currently, few predictive factors are used in routine practice outside of PDL1 and mutational status. The benefit of antiPD1/PDL1 agents increased with higher PDL1 expression (9), and is significantly lower in cases of some oncogenic driver mutations (such as EGFR or ALK) (10). Further molecular markers must be assessed in order to offer a better selection to patients. The potential biomarkers in development are discussed in this series (11).

There are many other questions regarding immunotherapy effectiveness or tolerance in NSCLC. This series of articles addressed two major questions regarding the interaction between radiotherapy and immunotherapy (Spaas and Lievens) and the possibility of administering immunotherapy in patients with autoimmune disorders (Coureau et al.). Radiosensitization through systemic therapy is achieved for conventional CT with platinum derivatives, while other drugs (such as anthracyclins or gemcitabine) must be avoided or used with major caution due to excessive toxicity during (or after) concurrent administration with radiotherapy. The interaction and potential increased toxicity of immuno-radiotherapy is a main scientific question with important impacts in routine practice, while the radiosensitization effect of immunotherapy remains debatable. At the other end, there is some evidence regarding an abscopal effect of radiotherapy leading to reinforced immunotherapy activity. All these subjects are discussed in the third manuscript of the series (Spaas and Lievens).

Finally, clinical trials denied inclusion of patients with autoimmune disorders. An *ad-litteram* implementation of phase III results could exclude these patients from potential effective therapy. Old retrospective series mainly dealing with melanoma suggested that giving immunotherapy in this situation risks activating the autoimmune disease as well as more immune adverse events (12). However, not all autoimmune diseases are equivalent in terms of reactivation or complication. The same could be said when considering stable diseases not requiring immunosuppressive drugs vs. those needing immunosuppression. The last manuscript of the series reports on the current evidence on this topic (Coureau et al.).

Even after all these improvements, non-small cell lung cancer remains a devastating tumor with dismal prognosis. The discovery of oncogenic driver mutations and immune checkpoints led to the introduction of the therapeutic armamentarium of very active drugs. Prognosis of advanced and metastatic NSCLC dramatically improved during the last 10 years, at least for some populations. Better defining the patients susceptible to receiving a high benefit from these new therapies as well as understanding the resistance mechanisms are research strategies that potentially will modify the therapeutic landscape in the near future.

## Author Contributions

All authors listed have made a substantial, direct, and intellectual contribution to the work and approved it for publication.

## Conflict of Interest

TB is consultant for InhaTarget, participated to Advisory Board for Bayer, Janssen, and Roche and is investigator for Pfizer, Merck, Astra Zeneca, Novartis, Peregrine, Amgen. The remaining authors declare that the research was conducted in the absence of any commercial or financial relationships that could be construed as a potential conflict of interest.

## Publisher's Note

All claims expressed in this article are solely those of the authors and do not necessarily represent those of their affiliated organizations, or those of the publisher, the editors and the reviewers. Any product that may be evaluated in this article, or claim that may be made by its manufacturer, is not guaranteed or endorsed by the publisher.
